# Clinicopathological mismatching in a patient presenting with clinical symptoms of Parkinson’s disease

**DOI:** 10.1002/alz.088164

**Published:** 2025-01-03

**Authors:** Eun Joo Chung

**Affiliations:** ^1^ Dementia and Neurodegenerative Disease Research Center, Inje University, St Charles, Korea, Republic of (South); Department of Neurology, Busan Paik Hospital, Inje University College of Medicine, Busan, Korea, Republic of (South); Inje University College of Medicine, BUSAN Korea, Republic of (South)

## Abstract

**Background:**

Because of clinically overlapping parkinsonian motor symptoms, it is hard to diagnose a specific disease in atypical parkinsonism or differentiate Parkinson’s disease (PD) from atypical parkinsonism. Herein, we report the clinicopathological mismatching of an autopsy‐confirmed PSP in a patient clinically diagnosed with PD.

**Method Clinical history:**

We reviewed the brief clinical history of a 70‐year‐old man and neurologic examination. We investigated the findings of the Mini‐mental state examination (MMSE), Clinical dementia rating (CDR), and Montreal cognitive assessment (MoCA). He performed brain magnetic resonance imaging (b‐MRI) and fluoro‐propyl‐carbomethoxy‐iodophenyl‐tropane (FP‐CIT)‐positron emission tomography (PET).

**Autopsy:**

The patient and his family gave antemortem consent for a postmortem examination. Autopsy was performed at the Inje University Brain Bank. The right brain was frozen and the left side was fixed into the 10% formalin. The fixed brain was photographed and coronally sliced at 1 cm intervals. After slicing, tissue block‐taking was done at 17 parts. Specimens were fixed with 10% buffered formalin at room temperature and embedded in paraffin. The hematoxylin‐eosin stain and immunohistochemical stains were performed.

**Result:**

The UPDRS motor scale of off‐state was 18.5, and the HY stage of off‐state was 2.0. The first MMSE and MoCA were 28, and the CDR score was normal. The third MMSE was assessed at 76 years old, and the score was 25 which increased by 2 points compared to a year ago. These results are in the table. The b‐MRI revealed mild small vessel ischemic change in the corona radiata (CR). FP‐CIT PET revealed asymmetrically bilateral decreased FP‐CIT uptake in both the putamen and caudate nucleus. The total brain weight of this patient was 1270mg. The neuropathological findings did not show the Lewy body pathologies. Instead, PSP pathology based on the tufted astrocytes was observed.

**Conclusion:**

His clinical symptoms were initially accordant with clinical diagnostic criteria for PD. However, neuropathological findings showed PSP pathology based on the tufted astrocytes. The patient had worse Parkinsonian motor symptoms, which pathologically suggested the brainstem‐predominant PSP rather than typical or cortical‐predominant.

